# EW-7197 eluting nano-fiber covered self-expandable metallic stent to prevent granulation tissue formation in a canine urethral model

**DOI:** 10.1371/journal.pone.0192430

**Published:** 2018-02-15

**Authors:** Kichang Han, Jung-Hoon Park, Su-Geun Yang, Deok Hee Lee, Jiaywei Tsauo, Kun Yung Kim, Min Tae Kim, Sung Gwon Gang, Dae-Kee Kim, Dong-Hyun Kim, Ho-Young Song

**Affiliations:** 1 Department of Radiology, Severance Hospital, Research Institute of Radiological Science, Yonsei University, College of Medicine, Seoul, Republic of Korea; 2 Department of Radiology, Research Institute of Radiology, Asan Medical Center, University of Ulsan College of Medicine, Seoul, Republic of Korea; 3 Department of Biomedical Engineering Research Center, Asan Medical Center, University of Ulsan College of Medicine, Seoul, Republic of Korea; 4 Department of Radiology, Northwestern University Feinberg School of Medicine, Chicago, IL, United States of America; 5 Department of New Drug Development and WCSL, Inha University College of Medicine, Incheon, Republic of Korea; 6 College of Pharmacy, Ewha Womans University, Seoul, Republic of Korea; Shanghai Jiao Tong University Medical School Affiliated Ruijin Hospital, CHINA

## Abstract

**Purpose:**

To evaluate an EW-7197-eluting nanofiber-covered stent (NFCS) for suppressing granulation tissue formation after stent placement in a canine urethral model.

**Materials and methods:**

All experiments were approved by the committee of animal research. A total of 12 NFCSs were placed in the proximal and distal urethras of six dogs. Dogs were divided into two groups with 3 dogs each. The control stent (CS) group received NFCSs and the drug stent (DS) group received EW-7197 (1000 μg)-eluting NFCSs. All dogs were sacrificed 8 weeks after stent placement Histologic findings of the stented urethra were compared using the Mann-Whitney *U* test.

**Results:**

Stent placement was technically successful in all dogs without procedure-related complications. On urethrographic analysis, the mean luminal diameter was significantly larger in the DS group than in the CS group at 4 and 8 weeks after stent placement (all *p* < 0.001). On histological examination, mean thicknesses of the papillary projection, thickness of submucosal fibrosis, number of epithelial layers, and degree of collagen deposition were significantly lower in the DS group than in the CS group (all *p* < 0.001), whereas the mean degree of inflammatory cell infiltration was not significantly different (*p* > 0.05).

**Conclusion:**

The EW-7197-eluting NFCS is effective and safe for suppressing granulation tissue formation after stent placement in a canine urethral model.

## Introduction

Placement of a covered self-expandable stent (SEMS) is a well-established method for the treatment of luminal strictures involving various nonvascular organs [1–5]. However, these stents exert constant mechanical stress to the adjacent tissue, resulting in granulation tissue formation. This is a major drawback of stent placement because granulation tissue formation can cause restenosis and recurrence of symptoms. Stent placement in the urethra is not an exception; therefore, granulation tissue formation is widely recognized as one of the most important obstacles to long-term stent placement in the urethra [5–8].

There is a growing understanding of the molecular mechanism of granulation tissue formation, and transforming growth factor-β1 (TGF-β1) has emerged as a key component in the proliferation of granulation tissue [9,10]. The desire to block the TGF-β1 signaling pathway has led to the development of several small-molecule drugs that act by inhibiting TGF-β1 from binding to its receptors [11,12]. Of these, EW-7197 is a novel oral TGF-β type-1 receptor kinase inhibitor [13,14]. This drug has been shown to have strong antifibrotic properties in animals [15,16] and the development of a drug-eluting stent using EW-7197 may be an attractive alternative for suppressing granulation tissue formation after stent placement in the urethra. Nanofiber meshes are flexible and highly porous meshes that is suitable for coating metallic stents and providing local drug delivery [17]. Recently, we have developed an EW-7197-eluting nanofiber-covered stent (NFCS). We hypothesized that this stent would suppress granulation tissue formation after stent placement in the urethra. Therefore, the purpose of this study was to evaluate EW-7197-eluting NFCSs for suppressing granulation tissue formation after stent placement in a canine urethral model.

## Materials and methods

### EW-7197-eluting nanofiber-covered stent

#### Stent construction

**Fig 1:**
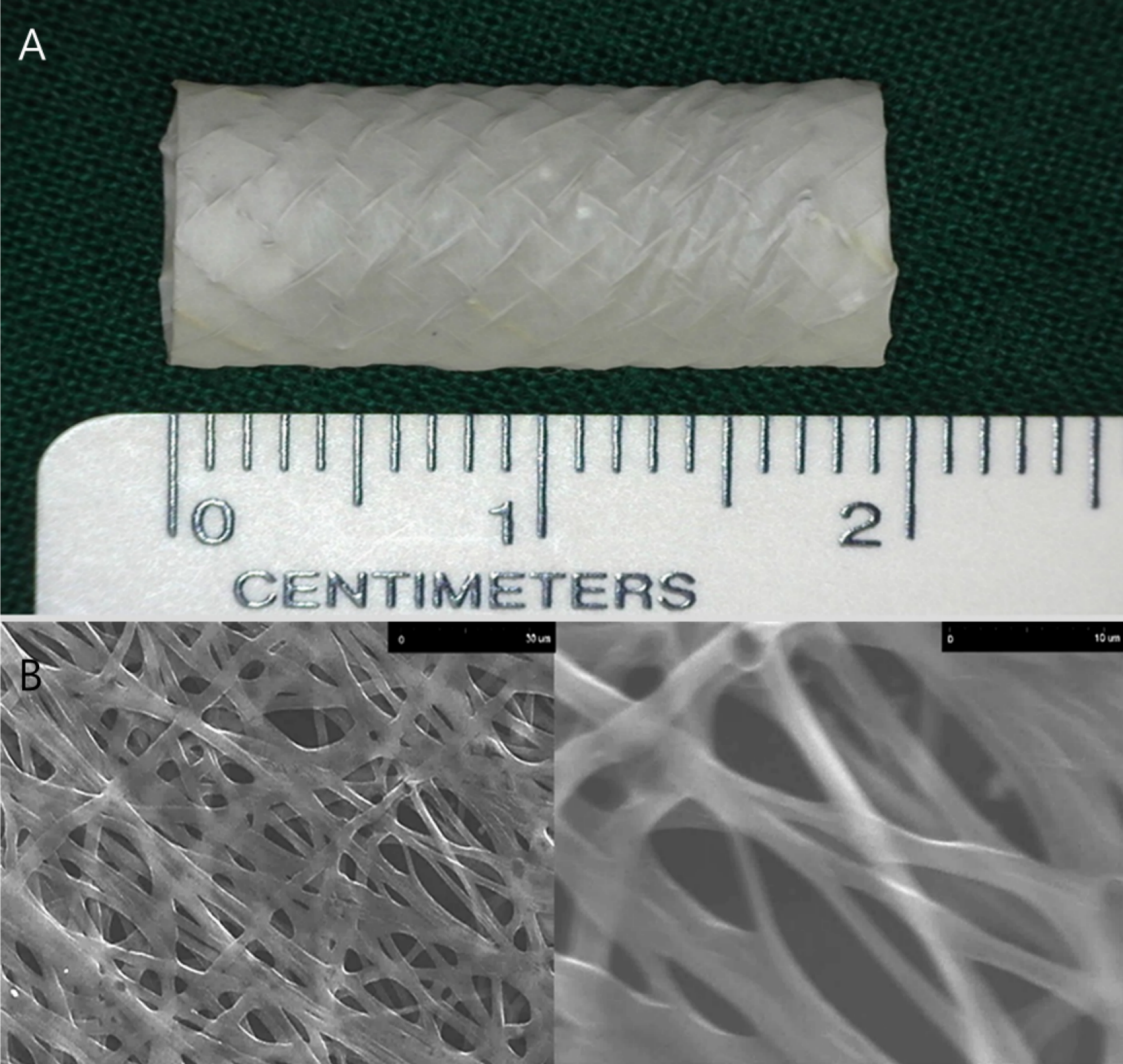
Representative optical images of (A) Nanofiber-covered, self-expandable metallic stent and (B) scanning electron micrographs of EW-7197 loaded nanofibers. The scale bars are 30 μm and 10 μm.

The stent used in this study was woven six times into a tubular configuration with six bends from a single thread of 0.15-mm-thick nitinol wire filament (Fig 1A). When fully expanded, the stent had a diameter of 10 mm and a length of 20 mm. Two radiopaque markers were attached to the ends of the stent to facilitate stent placement. The stent was made to our specifications by a local manufacturer (S&G biotech, Seongnam, Korea).

#### EW-7197-eluting nanofiber coating over the stent

The nanofiber covering membrane [drug-eluting poly-DL-lactic acid (PDLLA) and polyurethane (PU) backing layers] used in this study was constructed using electrospinning techniques. The electrospun scaffolds were constructed on the surface of the rolling stent and vacuum dried at room temperature for 24 h. PU (10 w/v%) and solvent polymer (90 w/v%) as a core was dissolved in tetrahydrofuran/dimethylacetamide (2:1) solution and then electrospun from the syringe at a rate of 0.2 ml/h. PDLLA (3.3 w/v%) and EW 7197 (20 w/v%) as a shell was sprayed from the syringe at a rate of 0.73 ml/h at the same time. The stents were rotated at 300 rpm and increased to 500 rpm over 2–4 h. Each EW-7197-eluting NFCS contained 1000 μg of EW-7197. A nanofiber layer on the surface of the stent was examined with a scanning electron microscope (SEM; TM-1000; Hitachi, Krefeld, Germany). The electrospun layer exhibited an apparent nanofiber structure, as shown in Fig 1B.

#### *In vitro* release study of EW-7197 nanofiber membrane

EW-7197 nanofiber membrane was placed inside a 50-mL conical tube with 50 mL of release medium, 10-mM PBS buffer of pH of 7.4 (n = 6). The tubes were placed in a shaking incubator at 37°C and 50 rpm. The release medium in each tube was collected at the predetermined time for further analysis, replaced with fresh media, and the concentration of EW-7197 was determined using a (high performance liquid chromatography) HPLC system (Agilent 1200 series, USA). The HPLC analysis was performed as follows. The composition of mobile phase was 71% water and 29% acetonitrile with 0.1% trifluroacetic acid. The flow rate was 1 mL/min throughout the 10-min run at 40°C. Chromatography was performed at 40°C and the eluent was monitored at a wavelength of 260 nm.

#### Animal study

The Institutional Animal Care and Use Committee at Asan medical center approved this study, which was conducted in accordance with the United States National Institutes of Health Guidelines on Humane Care and Use of Laboratory Animals.

Six male mongrel dogs (weight range, 20–25 kg; Orient Bio, Seongnam, Korea) were used for this study. A total of 12 NFCSs were placed in the proximal and distal urethras of six dogs. Dogs were divided into two groups with 3 dogs each. The dogs in the control stent (CS) group received non-drug eluting NFCSs in the proximal and distal urethras and the dogs in the drug stent (DS) group received EW-7197 (1000 μg)-eluting NFCSs in the proximal and distal urethras. All dogs were maintained in an independent cage (width: 1150 mm, depth: 1430 mm, and height: 2210 mm) made of stainless steel and they were allowed to socialize with animal care staff when the cage was cleaned twice a day. All dogs were supplied with food and water and mean room temperature was kept at 24°C with a 12-hour day-night schedule, which was fully automatically controlled. After stent placement, daily diet consumption, weight change, and behavioral change were monitored on a weekly basis. At 8 weeks after stent placement, dogs were sacrificed for histologic analysis. None of the dogs died prior to the study endpoints.

#### Stent placement

General anesthesia was induced by intra-muscularly administered ketamine hydrochloride (Yuhan, Seoul, Korea) and atropine sulfate (Daewon, Seoul, Korea) and was maintained with intravenously administered ketamine hydrochloride. After disinfecting the external urethral orifice with 0.05% chlorhexidine (Daewoong, Seoul, Korea), the urethra was lubricated using 2% lidocaine jelly (Dongsan, Ansan, Korea). A 180-cm guide wire (Radiofocus M; Terumo, Tokyo, Japan) was then inserted through the urethra and advanced into the urinary bladder. Retrograde urethrography (RGU; Omnipaque 300; GE Healthcare, Cork, Ireland) was performed using a straight 5F graduated catheter (Cook, Bloomington, Indiana) to determine the location of stent placement. Under fluoroscopic guidance (Artis Zee Multipurpose; Siemens, Muenchen, Germany), a stent-delivery system, consisting of a 10F sheath, a guiding olive tip, and a pusher catheter, was passed over the guide wire and advanced to the middle portion of the proximal urethra. The pusher catheter was then held in place with one hand while the sheath was slowly withdrawn in a continuous motion with the other hand until the NFCS was deployed. The stent-delivery system was then removed with the guide wire left in place, and another NFCS was placed in the middle portion of the distal urethra using the same technique. Antibiotics (penicillin, 800,000 U/day) and analgesics (ketoprofen, 1 ml/5 kg) were used for a period of three days after stent placement.

#### Urethrographic examination

In all dogs, RGU was performed in right anterior oblique projections with use of a straight 5F graduated catheter (Cook, Bloomington, Indiana) immediately after stent placement to verify position and patency of the stent. Follow-up RGU was performed 4 and 8 weeks later, and the luminal diameter was measured with the use of calibrated catheter. Software (Photoshop, version 6.0; Adobe Systems, Palo Alto, Calif) was used to acquire digital measurements of the inner luminal diameter of the stented urethra at three different levels. Measurements were repeated three times at each level, yielding an average value per level, and these values were subsequently averaged to obtain an overall average diameter of the segment. Analyses of the urethrographic findings were performed on the basis of the consensus of three observers blinded to the study.

#### Histological examination

**Fig 2:**
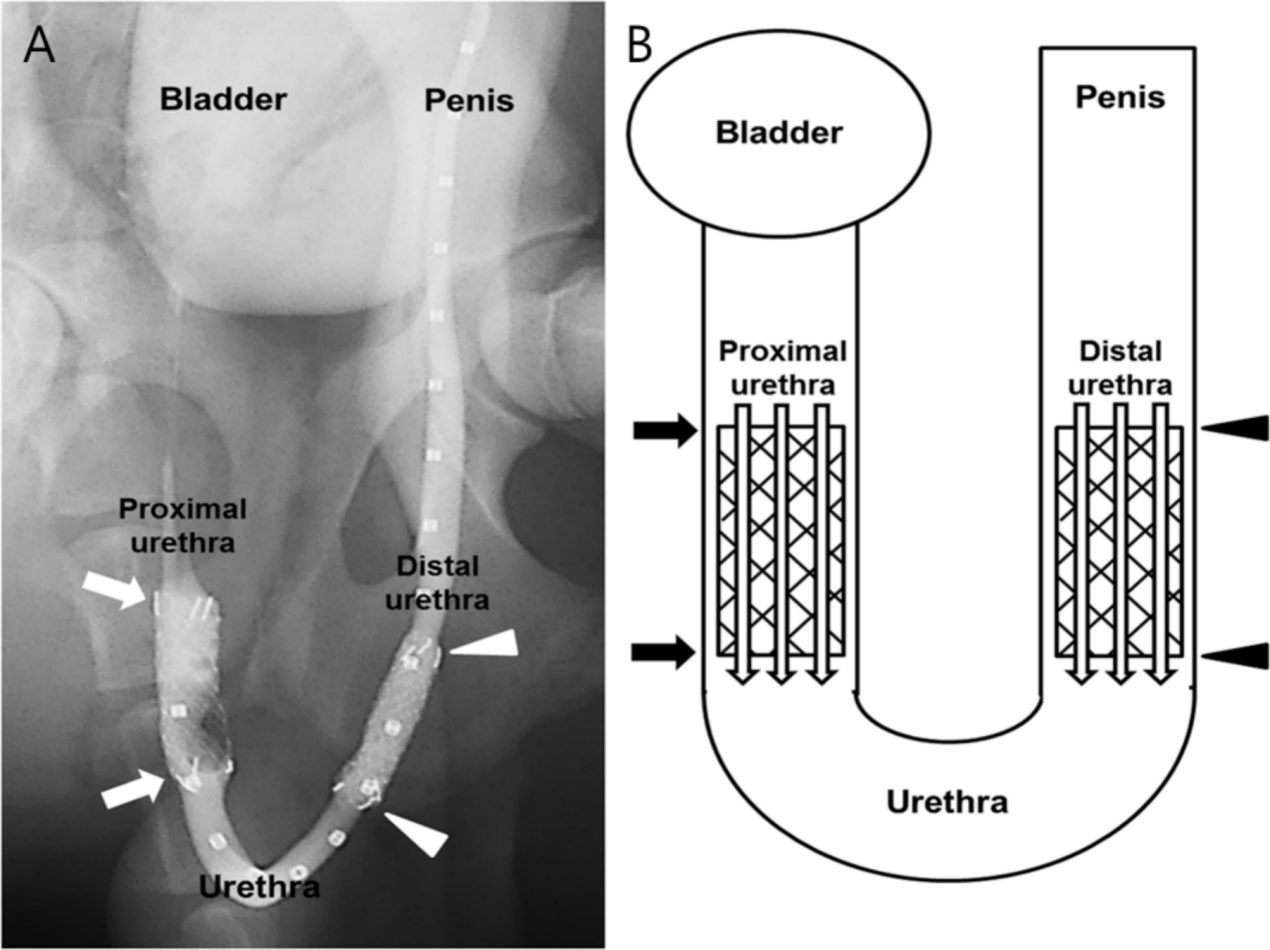
Locations of tissue sampled for histological examination. The canine urethra consists of the proximal pelvic urethra (arrows) and the distal cavernous urethra (arrowheads). (A) Retrograde urethrography of the canine urethra immediately after stent placement. (B) Schematic image showing the locations of tissue samples where a stent was present.

All dogs were sacrificed by means of administration of an overdose of xylazine hydrochloride (Rompun; Bayer, Seoul, Korea) 8 weeks after stent placement. The urethral tissue samples were fixed in 10% neutral-buffered formalin for 24 h. Fixed tissue samples were longitudinally sectioned at the three different portions of the segment with the stent (Fig 2). Paraffin blocks were prepared from the tissue samples and 5-μm-thick sections were obtained with a MR2258 microtome (HostoLine, Pantigliate, Italy). The slides were stained with hematoxylin and eosin (H&E) and Masson’s trichrome (MT) stains. Histological examination after H&E staining included determining the degree of submucosal inflammatory cell infiltration, the number of epithelial layers, the thickness of submucosal fibrosis, and the thickness of papillary projection. The degree of inflammatory cell infiltration was subjectively determined according to the distribution and density of the inflammatory cells (graded as 1, mild; 2, mild to moderate; 3, moderate; 4, moderate to severe; and 5, severe). The average values for the degree of inflammatory cell infiltration, the number of epithelial layers, the thickness of submucosal fibrosis, and the thickness of papillary projection were obtained by averaging eight points [18–20]. The degree of collagen deposition was determined on MT stained sections. Degree of collagen deposition was subjectively determined where 1 = mild, 2 = mild to moderate, 3 = moderate, 4 = moderate to severe, and 5 = severe. Histological examination of the urethra was performed with a BX51 microscope (Olympus, Tokyo, Japan). Measurements were obtained using Image-Pro Plus software (Media Cybernetics, Silver Spring, MD, USA). Analyses of the histological findings were performed on the basis of the consensus of three observers blinded to the study.

#### Statistical analysis

Data are expressed as the mean ± standard deviation. Comparisons between two groups were performed using the Mann–Whitney test. A *p* value of <0.05 indicates statistical significance. Statistical analyses were performed using SPSS software (version 22.0; SPSS, IBM, Chicago, IL, USA).

## Results

### *In vitro* release study

**Fig 3:**
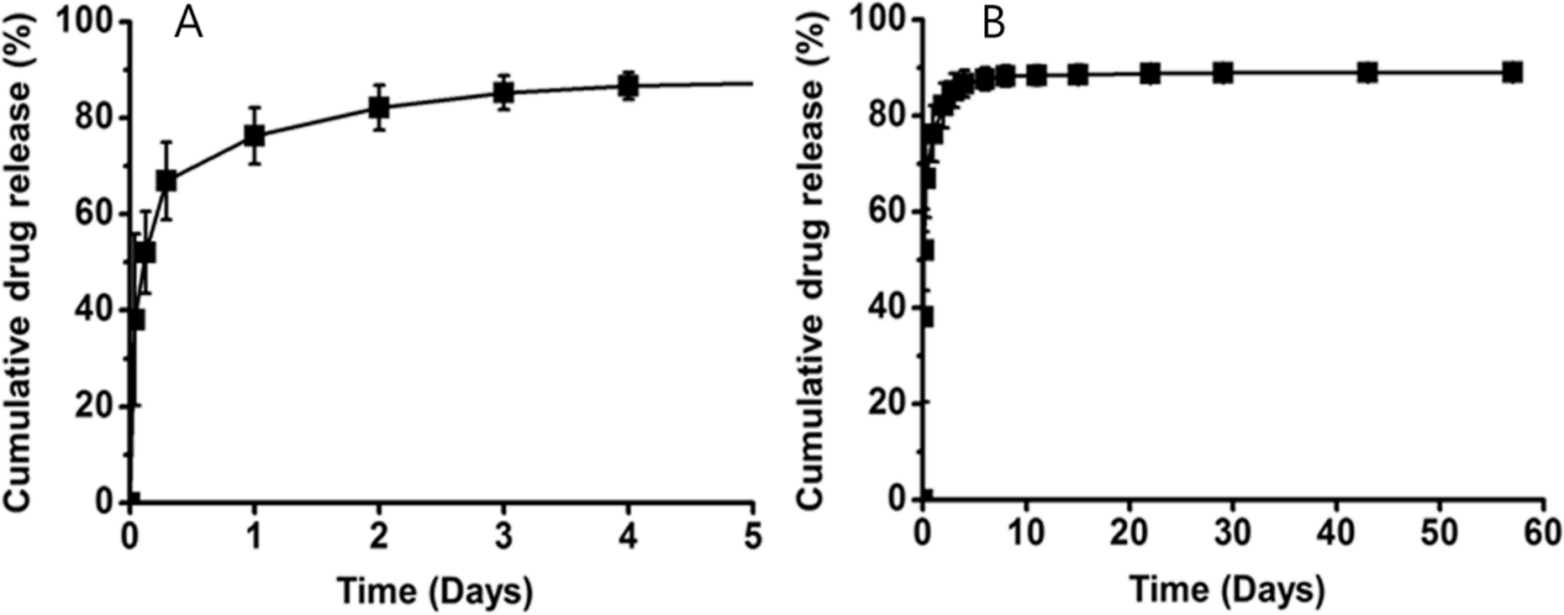
Curves show *in vitro* release profiles of EW-7197 from nanofiber membrane at (A) 5 days and (B) 60 days. Error bars = standard deviation of mean (n = 6).

To evaluate the drug release behavior of the PU (core)—PLLLA (shell) stents, *in vitro* drug release was monitored for 60 days using an HPLC system. As shown in Fig 3, approximately 80% of the encapsulated EW-7197 was released from the stents within 1 day, and the remaining encapsulated EW-7197 was slowly released thereafter.

### Stent placement

Stent placement was technically successful in all dogs without complications. A small amount of hematuria occurred immediately after stent placement in all dogs, but it subsided spontaneously. All animals survived until the end of the study without stent-related complications.

### Urethrographic findings

**Fig 4:**
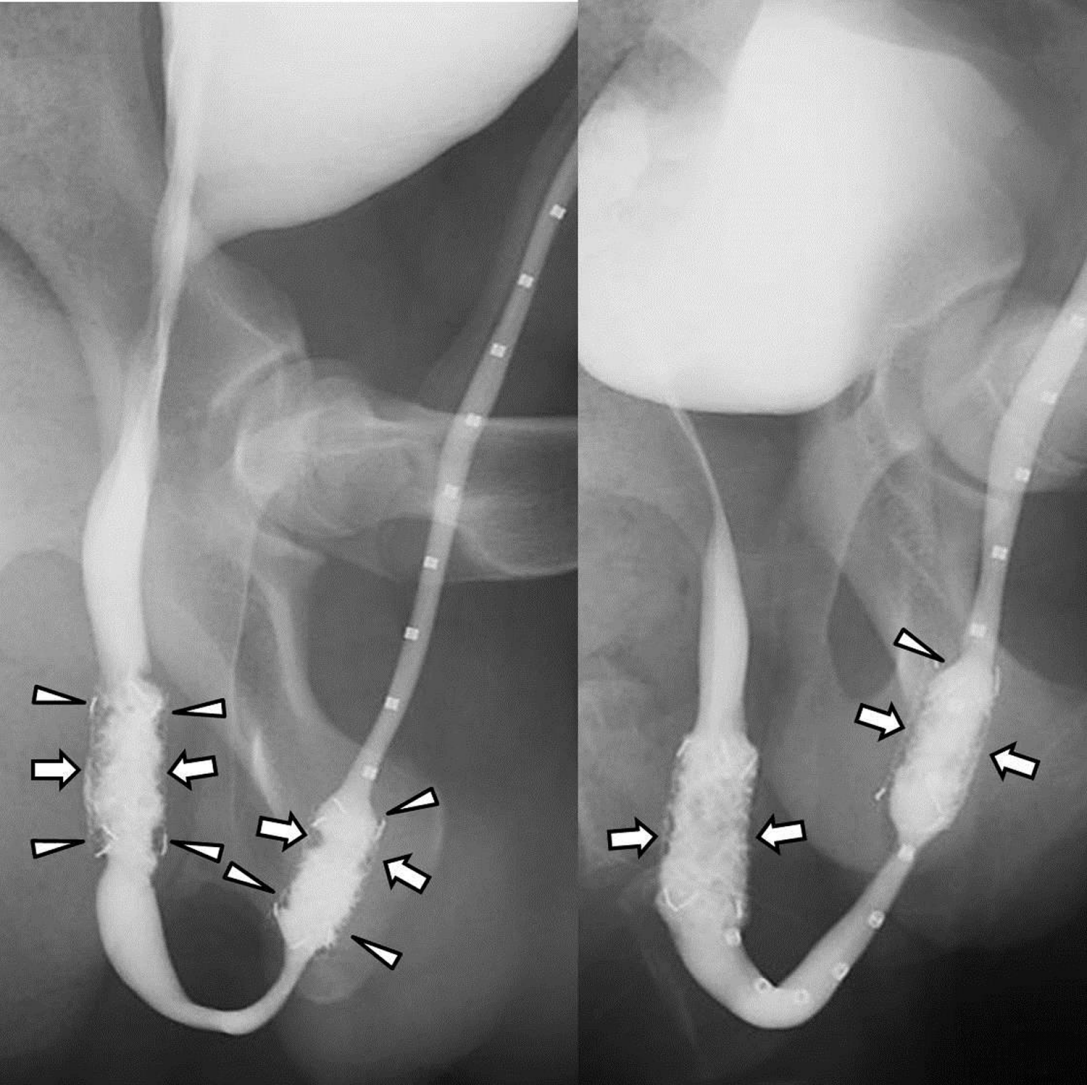
Retrograde urethrography images obtained 8 weeks after stent placement in dogs. (A) Image obtained after control stent placement shows filling defects (arrowheads) at both ends of the stents and in-stent stenosis (arrows) resulting from granulation tissue formation. (B) Image in drug stent group shows mild in-stent restenosis (arrows) in the proximal and distal stented urethras (arrows). At both ends of the stents, no definite filling defects were seen.

The urethrographic findings are summarized in Table 1 and are presented in Fig 4. At 4 weeks, the luminal diameter of the proximal stented urethra in the DS group was significantly larger than the CS group (*p* < 0.001). There was also a significant difference between the DS and CS groups (*p* = 0.002) in the luminal diameter of the distal stented urethra. The overall luminal diameter of the stented urethra in the DS group was significantly larger than the CS group (*p* < 0.001). At 8 weeks, the luminal diameter of the stented urethra in the DS group was significantly larger than the CS group for both the proximal (*p* < 0.001) and distal urethras (*p* = 0.015).

**Table 1:** Retrograde urethrographic findings after stent placement in canine urethra.

Location	4 weeks follow-up		*p* value	8 weeks follow-up		*p* value
	CS group	DS group		CS group	DS group	
**Proximal**	7.36 ± 1.51	9.20 ± 1.74	<0.001	6.68 ± 1.39	8.60 ± 1.37	<0.001
**Distal**	6.62 ± 1.32	8.31 ± 1.57	0.002	6.20 ± 1.05	7.72 ± 1.58	0.015
**Total**	6.99 ± 1.45	8.67 ± 1.66	<0.001	6.51 ± 1.27	8.16 ± 1.46	<0.001

**Note.** Data are mean diameters of the six stented urethra in millimeters ± standard deviations. CS; control stent, DS; drug stent

### Histological findings

**Fig 5:**
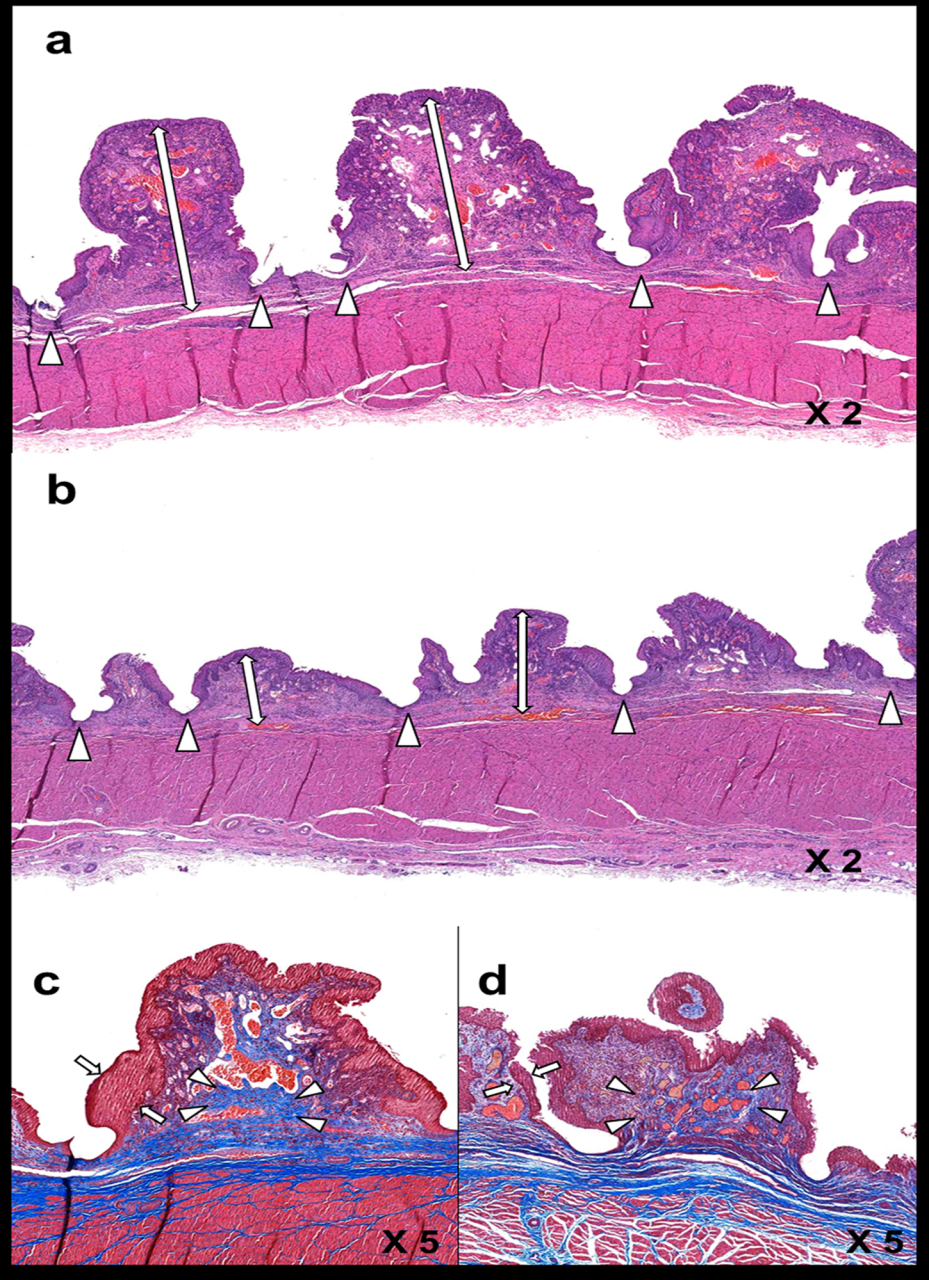
Representative microscopic images (hematoxylin and eosin staining magnification × 2 [A,B] and Masson’s trichrome staining magnification × 5 [C,D]) of histological sections at 8 weeks after stent placement. The thickness of papillary projection (arrows) was significantly increased in the CS group (A) compared to the DS group (B). Arrowhead = stent struts (A,B). The degree of collagen deposition (arrowheads) was significantly greater in the CS group (C) than the DS group (D). The number of epithelial layers (arrows) in the CS group (C) was significantly higher than the DS group (D). CS: control stent, DS: drug stent.

The histological findings are presented in Table 2, and examples are shown in Fig 5. The overall mean thickness of the papillary projection was significantly less in the DS group than in the CS group for both the proximal and distal stented urethra (all *p* < 0.001). The mean thickness of submucosal fibrosis (all *p* < 0.001) and the mean number of epithelial layers (all *p* < 0.001) were also significantly less in the DS group than in the CS group for both the proximal and distal stented urethra. The mean degree of inflammatory cell infiltration did not significantly differ between the CS and DS groups (*p* = 0.480; proximal urethra, *p* = 0.484; distal urethra, *p* = 0.742). The mean degree of collagen deposition was significantly less in the DS group than in the CS group for both the proximal and distal urethras (all *p* < 0.001). The comparison between the proximal and distal urethras did not show significant differences for any variables between groups (all *p* > 0.05).

**Table 2:** Histological findings after stent placement in canine urethra.

	Location	CS group	DS group	*p* value
**Thickness of Papillary Projection (mm)**	**Proximal**	2.67 ± 0.48	1.11 ± 0.28	<0.001
	**Distal**	2.78 ± 0.56	1.26 ± 0.35	<0.001
	**Total**	2.72 ± 0.52	1.19 ± 0.31	<0.001
**Thickness of Submucosal Fibrosis (mm)**	**Proximal**	1.27 ± 0.55	0.67 ± 0.11	<0.001
	**Distal**	1.33 ± 0.64	0.64 ± 0.12	<0.001
	**Total**	1.30 ± 0.58	0.66 ± 0.11	<0.001
**Number of Epithelial Layers**	**Proximal**	4.84 ± 0.76	3.56 ± 0.72	<0.001
	**Distal**	5.16 ± 0.88	3.44 ± 0.72	<0.001
	**Total**	5.00 ± 0.83	3.50 ± 0.71	<0.001
**Degree of Inflammatory Cell Infiltration**	**Proximal**	3.28 ± 0.77	3.16 ± 0.72	0.506
	**Distal**	3.13 ± 0.66	3.06 ± 0.84	0.742
	**Total**	3.20 ± 0.72	3.11 ± 0.78	0.480
**Degree of Collagen Deposition**	**Proximal**	4.41 ± 0.67	3.34 ± 0.75	<0.001
	**Distal**	4.44 ± 0.62	3.44 ± 0.76	<0.001
	**Total**	4.42 ± 0.64	3.39 ± 0.75	<0.001

Note. Data are mean ± standard deviation. CS; control stent, DS; drug stent

## Discussion

Placement of SEMSs for benign and malignant strictures of various organs has been around for several decades [1–5]. The downside of this treatment is restenosis resulting from granulation tissue formation through the mesh or around the edges of the stents [21]. Deeper understandings into the pathophysiology of granulation tissue formation and advances in stent technology have expanded their clinical applications. There have been several efforts to use drug-eluting stents in the treatment of strictures in nonvascular luminal organs [22–24]. The benefits of stent-based drug delivery are to maximize the level of therapeutic agents in local tissue, lower drug dosage, and minimize systemic toxicity. In the present study, we designed EW-7197-eluting covered stents and tested their efficacy in suppressing granulation tissue formation in an animal model. Our results suggest that EW-7197 locally delivered by a stent successfully reduces or inhibits granulation tissue formation secondary to stent-induced mechanical injury in a canine urethral model, as evidenced by significantly larger luminal diameter on RGU and less granulation tissue formation on histological examination in the DS group than in the CS group.

TGF-β and its receptors are uniformly overexpressed in fibrotic diseases of various organs, and it has been reported to be a potent, profibrogenic cytokine resulting in pathologic fibrosis [9,25]. Therefore, the signaling pathway for fibrosis via TGF-β has been a promising target for drug development. Various attempts to block the TGF-β pathway have been made and EW-7197 is a recently introduced TGF-β type-1 receptor kinase inhibitor [13]. The safety and efficacy of EW-7197 has been documented in animal models, and several investigators have reported that EW-7197 safely inhibited hepatic, renal, and pulmonary fibrosis and stent-induced granulation tissue formation by blocking the TGF-β/Smad and/or reactive oxygen species (ROS) signaling pathway [15,16,26]. As stent-induced benign stricture appeared to be subject to TGF-β, EW-7197 was used as an antifibrotic agent in the present study.

In the current study, granulation tissue formation based on the luminal diameter of the stented urethra and histological examination was significantly decreased in the DS group compared to the CS group in both the proximal and distal urethras. In a study by Shin et al., they placed the paclitaxel-eluting covered stents into canine urethras and reported that decreased granulation tissue formation was observed only in the proximal urethra [20]. They postulated that the distal urethra, with a smaller diameter, was less expandable and exposed to higher mechanical microtrauma than in the proximal urethra. As a result, granulation tissue formation was thought to be more prominent in the distal urethra. Despite the different anatomic milieu between the proximal and distal urethras, granulation tissue formation was uniformly suppressed in the DS group in this study. The possible reason for the discrepancy is that EW-7197 has a more potent antifibrotic effect than paclitaxel for overcoming the harsher environment in the distal urethra.

On histological examination, the mean thicknesses of papillary projection, thickness of submucosal fibrosis, number of epithelial layers, and degree of collagen deposition were significantly less in the DS group than in the CS group, whereas inflammatory cell infiltration was not different. Given the key role of TGF-β in fibrosis after stent-induced mechanical injury, a previous study demonstrated that oral administration of EW-7197 after bare metallic stent placement successfully inhibited granulation tissue formation by blocking the TGF-β signaling pathway in a rat esophageal model [15]. Although inflammatory cells may be continuously recruited by an indwelling stent acting as the stimulus, EW-7197 appears to prohibit the inflammatory phase from progressing to the proliferative phase [10]. In a study by Kim et al., they placed bare metallic stents into rat urethra and investigated the efficacy of intraperitoneal administration of IN-1233, another TGF-β type-1 receptor kinase inhibitor [27]. Their outcomes were consistent with the present study, except for inflammatory cell infiltration, which was significantly increased in the drug group in their study. EW-7197 was a newer TGF-β type-1 receptor kinase inhibitor class drug and did not seem to stimulate inflammatory cell infiltration that had been seen in IN-1233. As the accumulation of inflammatory cells may increase the likelihood of subsequent granulation tissue formation, EW-7197 is assumed to be a better candidate for drug-eluting stents.

Nanofiber coating by means of electrospinning was attempted to overcome the initial burst of the drug-eluting stents that was observed with PU-covered stents [20,22,28]. Surprisingly, the *in vitro* release study demonstrated that approximately 80% of the drug was eluted from the stents within 1 day after which the elution rate slowed down and reached a plateau after 10 days. This was in marked contrast to the sustained drug delivery from the NFCS in a previous report [17]. Despite the unexpected in vitro findings, our current EW-7197-eluting NFCS is able to suppress granulation tissue formation after stent placement. We have two theories for the substantial release of the drug at an early phase. As the *in vitro* pharmacokinetic properties of EW-7197 may differ significantly from those in in vivo settings, including the low pH of the canine urethra, there is a possibility that the excellent outcome resulted from a slower but steadier in vivo release of the drug. This hypothesis warrants further studies to see if the similar drug elution pattern can be acquired in the *in vivo* environment. The other assumption is that the anti-fibrogenic effect of EW-7197 was so potent that granulation tissue formation was suppressed in a prolonged fashion even with the initial burst of about 80% of EW-7197. This encourages more research on whether EW-7197 is effective in inhibiting fibrosis with 1 or 2 days of administration rather than sustained drug release.

On RGU, filling defects and luminal irregularities due to granulation tissue formation were seen at both ends of the stent as well as the stented urethra. This was likely attributable to the partial degradation of the covering membrane. The acidic environment of the urethra might have taken its toll on the PDLLA-PU complex covering membrane itself and led to the partial degradation. As the covering membrane hinders the granulation tissue from growing through the stent mesh, further studies are needed to develop a better EW-7197-eluting covering material that does not degrade.

There were some limitations in this study. First, although many of the variables of interest reached statistical significance, the sample size was too small to perform a robust statistical analysis. However, the differences in RGU and histological findings between the two groups were indisputable. Second, quantitative analyses for the expression levels of TGF-β and its mediators, such as Smad2/3, were not possible because the stents were placed in a canine urethra model. Third, we could not conduct *in vivo* pharmacokinetic studies on the EW-7197-eluting NFCS because of the small sample size and also did not evaluate in vitro degradation and mechanical properties of the PDLLA fibrous membranes. Given the unexpected *in vitro* drug release profile, *in vivo* pharmacokinetic studies are warranted. Third, some of the measurements in this study were obtained subjectively and quantitative data such as morphometric analyses or cell counting would have provided more reliable data. Finally, long-term effectiveness of the EW-7197-eluting NFCS was not evaluated. If granulation tissue had developed after 100% of the encapsulated EW-7197 had been released, it would have provided further evidence supporting the effectiveness of EW-7197 in inhibiting stent-induced granulation tissue formation.

In conclusion, the direct and local therapy with EW-7197 via a covered stent is effective and safe for suppressing granulation tissue formation after stent placement in a canine urethral model. The promising results in this study warrant further studies on this drug for broader applications for stents in other parts of the organs.

## Supporting information

S1 Table Data of the diameter of the proximal and distal canine urethras and the microscopic findings in both groups after stent placement. (XLSX)
